# Effective Synthesis of Highly Oxidized Graphene Oxide That Enables Wafer-scale Nanopatterning: Preformed Acidic Oxidizing Medium Approach

**DOI:** 10.1038/s41598-017-04139-0

**Published:** 2017-06-20

**Authors:** Chun-Hu Chen, Shin Hu, Jyun-Fu Shih, Chang-Ying Yang, Yun-Wen Luo, Ren-Huai Jhang, Chao-Ming Chiang, Yung-Jr Hung

**Affiliations:** 1 Department of Chemistry, National Sun Yat-sen University, Kaohsiung, 80424 Taiwan; 2 Department of Photonics, National Sun Yat-sen University, Kaohsiung, 80424 Taiwan

## Abstract

Demand for rapid and massive-scale exfoliation of bulky graphite remains high in graphene commercialization and property manipulation. We report a procedure utilizing “preformed acidic oxidizing medium (PAOM)” as a modified version of the Hummers’ method for fast and reliable synthesis of graphene oxide. Pre-mixing of KMnO_4_ and concentrated H_2_SO_4_ prior to the addition of graphite flakes enables the formation of effectively and efficiently oxidized graphene oxide (EEGO) featured by its high yields and suspension homogeneity. PAOM expedites diffusion of the Mn-oxidants into the graphite galleries, resulting in the rapid graphite oxidation, capable of oxidizing bulky graphite flakes (~0.8 mm in diameter) that can not be realized by the Hummers’ method. In the scale-up tests, ten-time amount of graphite can be completely exfoliated by PAOM without need of extended reaction time. The remarkable suspension homogeneity of EEGO can be exploited to deposit ultra-flat coating for wafer-scale nanopatterning. We successfully fabricated GO optical gratings with well-defined periodicity (300 nm) and uniform thickness (variation <7 nm). The combination of the facile and potent PAOM approach with the wafer-scale patterning technique may realize the goal for massive throughput graphene nanoelectronics.

## Introduction

Graphene oxide (GO) is touted as a viable route for the large-scale production and manipulation of graphene, a wonder material which has spawned a cornucopia of applications in high speed semiconductors, flexible devices, optoelectronics, and energy storage^1–4^. Among various GO synthesis protocols, the Hummers’ method is considered to be the best in terms of safety and productivity^5, 6^. The process involves treatment of flake graphite with concentrated H_2_SO_4_ as the intercalant to make the graphite swell, followed by conversion into the oxidized form with the oxidizing agent (KMnO_4_)^7, 8^. Upon dissolution in water, bulk graphite oxide can then exfoliate to individual dispersed GO sheets. In order to be more efficient, less defective and friendly to the environments, variations of the Hummers’ method have been continuously sought^5–9^.

To tailor the properties of pristine graphene, GO with sufficient quantity of oxygenated functional groups is desired because they provide reactive sites for chemical derivatization. However, pursuit of higher level of oxidation (high O/C ratio) has been rarely emphasized in current GO synthesis methods. In principle, the higher the oxidation degree of GO, the longer preparation time and the greater amount of oxidant are needed. We quest for a recipe which increases the O/C ratios beyond the existing limit in a reasonable timeframe without consuming an excessive amount of oxidants. Here we report a modified Hummers’ method by adding graphite flakes into a pre-mixed sulfuric acid, phosphorus pentoxide and potassium permanganate solution. Exposing graphite to such a “preformed acidic oxidizing medium” (PAOM) enhanced the oxidant-diffusion rate inside the graphite interlayers; therefore, the GO products gained elevated oxidation degrees within a short period of time. It is noteworthy that the resultant GO which we define as the effectively and efficiently oxidized graphene oxide (EEGO) was featured by a bright yellow color (due to the loss of electronic conjugation) reflecting its high degrees of oxidation^6^. As a contrast, the conventional Hummers’ method requires several hours and even days depending on the graphite flake size^10–12^. Because such effective oxidation weakened the van der Waals forces and facilitated the exfoliation process, EEGO was well dispersed in water permitting it to be uniformly casted onto substrates in the form of thin films. We demonstrate that the ability of EEGO to remain highly dispersive can lead to controllable fabrication of wafer-size superfine optical gratings. Although physical patterning of graphene into micro-stripes can be achieved by e-beam lithography, interference lithography, direct laser writing, and soft lithography^13–16^, none of the prior works achieved wafer-scale submicron grating pattern on graphene or its related materials.

## Experimental section

### EEGO preparation by PAOM

In a typical synthesis using PAOM method, concentrated H_2_SO_4_ (23 mL, 98%) was first mixed with P_2_O_5_ (1 g) and then KMnO_4_ (3 g) for 3 min. Graphite flakes (1 g, Alfa Aesar, 325 mesh) and NaNO_3_ (0.5 g) were ground together and added to the reaction mixture for 10 min. Afterwards, the reaction system was transferred to a 35 °C oil bath with vigorous stirring for 1 hour. DI water (10 mL) was added carefully for five times to avoid overheating due to the exothermic reaction. The resultant was then warmed up to 85 °C and maintained for 15 min. After cooling down to room temperature a slow addition of H_2_O_2_ (10 mL, 35%) was followed. A bright yellow color EEGO can be obtained. When the initial graphite amount was increased to 10 g with the proportional amounts of reagents of PAOM, the GO products (denoted as 10X-EEGO) can be produced with reaction time at 1 hr. No safety hazard issue was noticed during our tests of PAOM.

### GO preparation by Hummers’ method

The GO preparation using Hummers’ method was conducted following the original work reported by Hummers and Offeman in ref. 5. A concentrated H_2_SO_4_ (23 mL) was first stirred at 0 °C in an ice bath. A mixture of graphite flakes (1 g) and NaNO_3_ (0.5 g) was added to the concentrated H_2_SO_4_. Subsequently, KMnO_4_ (3 g) was then added slowly to prevent the reaction temperature higher than 20 °C. The resultant was then warmed to 35 °C with stirring for 30 min. After the DI water addition (46 mL), the mixture was heated in an oil bath and the overall reactant temperature was maintained at 98 °C for 15 min. Finally, the resultant was cooled down to room temperature followed by H_2_O_2_ (10 mL, 35%) addition. The products with the appearance of yellow-brown color were denoted as Hummers’ graphene oxide (HGO). The products with a dark green color were obtained and named as green-HGO (gHGO).

### Purification of Graphene Oxide

All the GO samples were washed and filtrated with HCl aqueous solution (37% HCl:DI water = 10:100 mL) several times, followed by dialysis in water to reach neutral pH conditions under aqueous environment. These GO samples were subjected to a 6000 rpm centrifuge for 10 min. The precipitates were only analyzed by XRD and the supernatants were collected for various analyses (see below).

### Graphene oxide grating preparation

A variety of one-dimensional (1D) graphene oxide (GO) gratings over 2-inch silicon wafers was produced by laser interference lithography and plasma/ozone etching. The silicon wafers are first cleaned by a sequence of acetone, isopropanol, and de-ionized wafer, and blown dry with nitrogen gas. A thin GO film was deposited on the cleaned silicon substrate by spin coating with an aqueous GO suspension solution at 3000 rpm for 30 seconds. The thickness of GO film ranges from 20 to 50 nm that can be controlled by the concentrations of aqueous GO suspension solutions (5 to 10 mg/mL). After baking the GO film at 100 °C for 60 seconds, a 70-nm-thick bottom antireflection coating (BARC) (Brewer Science iCON-7) layer was deposited atop the GO film by spin coating at 3000 rpm for 60 seconds followed by the baking process at 205 °C for 60 seconds. A 120-nm-thick positive photoresist layer (Allresist AR-P 3170) was deposited atop the BARC layer by spin coating at 4000 rpm for 60 seconds followed by the soft baking at 100 °C for 60 seconds. The samples were then transferred to the laser interference lithography system and exposed to interference fringes with an exposure dose of about 28 mJ/cm^2^. In this demonstration the grating periodicity was set to 300 nm, but in fact a flexible grating periodicity ranging from 190 nm to 3000 nm can be achieved by simply adjusting the incident angles of two laser beams. Finally, the samples were immersed in a diluted 2.38% tetramethylammonium hydroxide (TMAH) photoresist developer for 8 seconds to produce the grating structures.

We conducted the three-step etching process to transfer the grating patterns from the photoresist to the GO film. First the transfer of grating patterns into the BARC layer was carried out by anisotropic oxygen plasma etching under an oxygen flow of 20 sccm, a pressure of 80 mTorr, and a RF power of 100 W for 24 seconds. Afterward, the wafer is cleaned by a sequence of acetone, isopropanol, and de-ionized wafer and blown dry with nitrogen gas. This clean process aims to remove the residual photoresist on the wafer to decrease the aspect ratio of the grating patterns, and thus the pattern shrinkage during the following etching process can be reduced. Second, the transfer of grating patterns from BARC layer into the GO film was again carried out by the same anisotropic oxygen plasma etching for 14 seconds to form GO gratings. Finally, the residual BARC material atop the GO gratings was removed by ozone etching with an oxygen flow of 1 L/min under a lifted temperature environment of 135 °C for 30 minutes. The etching rate of BARC material is an order of magnitude higher than the etching rate of GO film so that complete removal of BARC material atop the GO gratings is attainable.

### Materials characterization

The Raman, X-ray photoelectron spectroscopy (XPS), scanning electron microscopy (SEM) and atomic force microscopy (AFM) samples were prepared by drop casting and drying the diluted ethanol GO suspension onto silicon oxide wafers. Raman spectra were carried out on a WITec Confocal Raman Microscope Alpha300R with a 532 nm laser. The XPS data were recorded with Kratos Axis Ultra DLD with Mg/Al achromatic source. SEM observations were conducted with a thermal field emission scanning electron microscope (FE-SEM, model: inspect F50 equipped with an energy-dispersive X-ray spectrometry (EDS). AFM results were measured by Veeco Multimode NanoScope IIIa in contact mode. The X-ray diffraction (XRD) and elemental analysis (EA) samples were oven dried powders from the GO suspension. The XRD patterns were obtained by Bruker D2 Phaser diffractometer with a Cu-Kα X–ray (λ = 1.5418 Å) radiation. EA data were acquired using Vario EL III. The UV-visible (UV-vis) and dynamic light scattering (DLS) samples were the aqueous GO suspension solutions. The UV-vis spectra were obtained using a V-600 UV–visible spectrometer in 0.06 mg/mL. DLS were collected on N5 Submicron Particles Size Analyzer. The cyclic voltammograms (CVs) were carried out with a conventional three-electrode configuration and a CHI614D electrochemical analyzer.

## Results and Discussion

**Figure 1:**
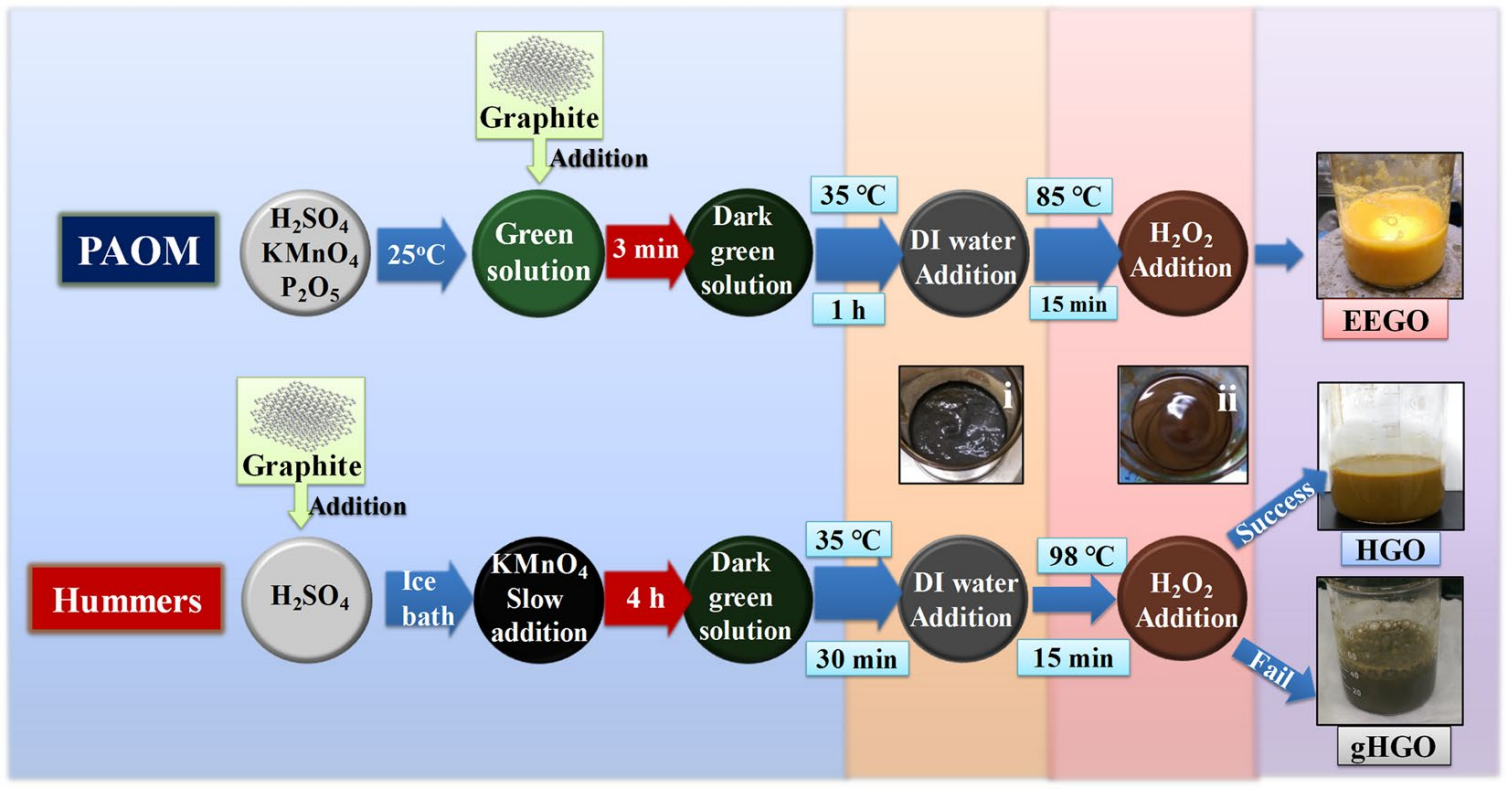
GO preparation by PAOM and conventional Hummers’ approaches. Photograph i and ii illustrate the physical appearance of the intermediate stages.

Detailed comparisons are made between PAOM and the benchmarking Hummers’ method. The major difference is the addition sequence of graphite in the reaction mixture. As illustrated in Fig. 1, we concoct the PAOM from KMnO_4_, H_2_SO_4_ and P_2_O_5_ prior to the graphite addition at room temperature, whereas the protocol of the Hummers’ requires slow feeding of KMnO_4_ to the slurry of graphite and H_2_SO_4_ in an ice-bath. The EEGO product prepared by PAOM displays the brightest yellow color among all the others, suggesting that graphite is subjected to substantial oxidation, and its production is proven to be reproducible. The total time for completing the PAOM procedure is shortened to just few hours which the Hummers’ approach cannot emulate. The GO obtained by the Hummers’ method (HGO) shows a brownish color, which might suggest a lower degree of oxidation. We also noticed that the HGO preparation is quite technically demanding. Any improper operation, particularly arbitrary addition rates of KMnO_4_, may result in another dark green product accompanied with black chunks (gHGO), which is considered to be the “failed” product characterized by the presence of visible solid residues due perhaps to the poor graphite oxidation and exfoliation. The 98 °C heating originated from the Hummers’ method can be reduced to a lower temperature of 85 °C, still giving the same products of EEGO. The incorporation of P_2_O_5_ in PAOM is aimed to reduce the water content and thus preserve the reactivity of the oxidants.

Because the acidic manganese-based oxidants, such as Mn_2_O_7_ formed during the synthesis (KMnO_4_ + 3H_2_SO_4_ → K^+^  + MnO_3_^+^  + H_3_O^+^  + 3HSO_4_^−^; MnO_3_^+^  + MnO_4_^−^ → Mn_2_O_7_)^7, 17, 18^, are known to be vulnerable to water molecules and elevated temperatures. The risk of a hasty KMnO_4_ addition into the graphite-H_2_SO_4_ mixture would result in a drastic increase of local temperature and releasing of H_2_O, both responsible for oxidant decomposition. The conventional method also suffers from incomplete dissolution of KMnO_4_, making the insertion and oxidation steps ineffective. As the diffusion of oxidants into graphite galleries governs the rate of oxidation^7^, the slow feeding of KMnO_4_ into H_2_SO_4_ (the Hummers’ method) leads to poor contact between graphite and the Mn-oxidants at the initial stage of oxidation. In stark contrast, the added graphite should encounter the Mn-oxidants right upon mixing with the homogeneous PAOM. The ready-established concentration gradient drives the oxidants to quickly fill up the graphite interlayers and short-time oxidation is thus achieved.

**Figure 2:**
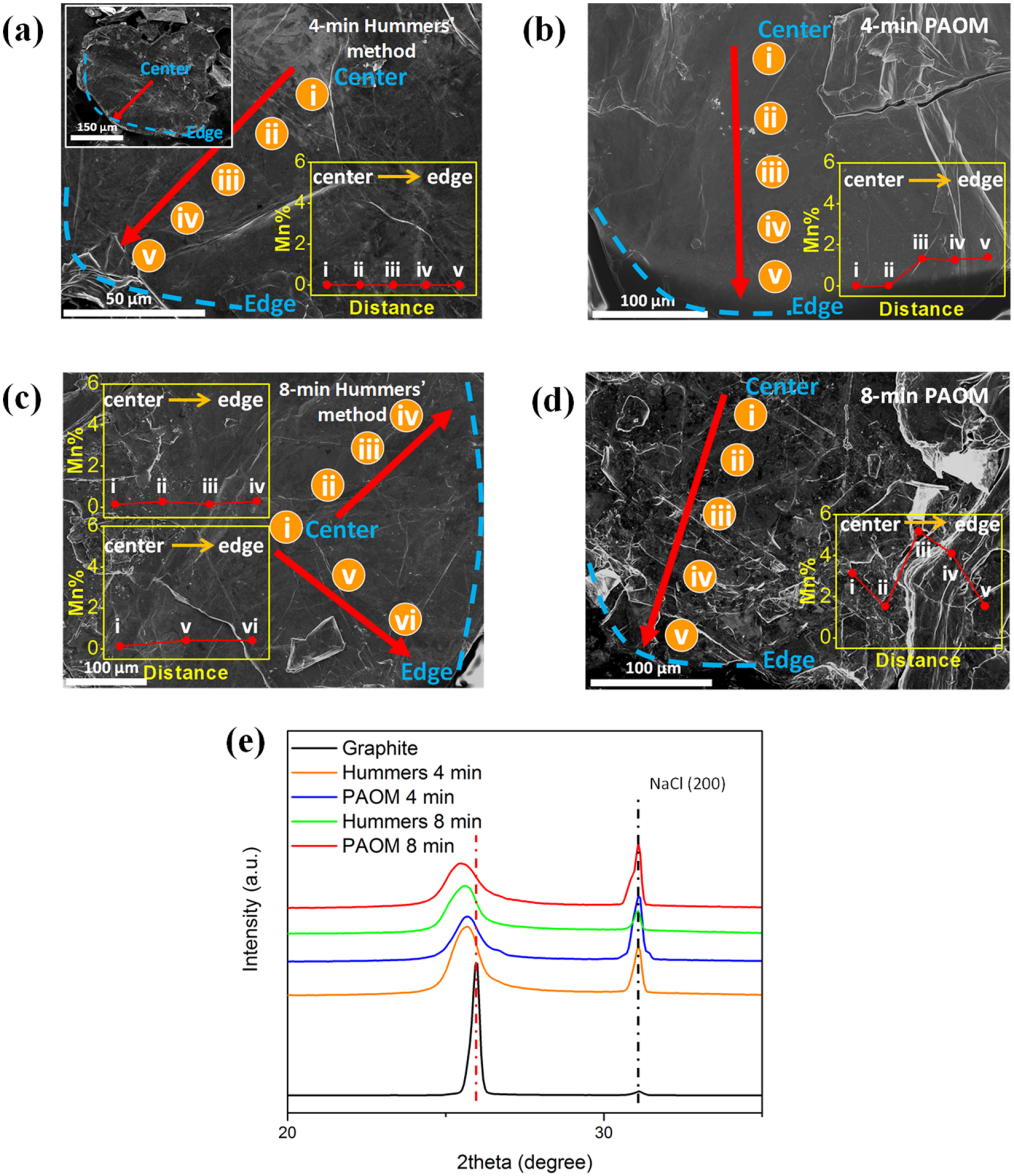
The extent of Mn-oxidant diffusion monitored by EDS in the partially reacted graphite by PAOM and conventional Hummers’ method. The red-arrows indicate the direction from the center toward outer edge (the blue dash-lines) of the individual graphite flake, as shown in the inset of (**a**). The Mn contents are normalized by carbon and summarized in the insets. (**e**) The XRD patterns of the partially reacted samples in (**a**)–(**d**) and pristine graphite. NaCl is the internal standard.

To consolidate this concept, we monitored the Mn-distributions by EDS from center to edge of the partially reacted graphite flakes isolated by quenching the synthesis at the 4 to 8 min time. Because EDS is not an surface sensitive technique, the source of characteristic X-ray covers a depth range of several microns below the graphite surface, equivalent to around 10,000 layers of graphite galleries. This argues that the EDS signals represent the distribution of Mn-oxidant in micron-scale depth, rather than coming from the superficial residue. To avoid ambiguous interpretation, we used a bulky graphite flakes with the average diameters of 0.8 mm (around 20-time larger than the 325-mesh graphite for EEGO synthesis) as the starting materials in this test (see Fig. S-1). The data (all the orange spots) were acquired stepwise from the center toward the outer edge of an individual graphite flake (see the inset of Fig. 2(a)). As shown in Fig. 2, pronounced Mn signals are present at the center of the samples prepared by PAOM within 8 min, while no appreciable Mn signals can be found in samples obtained from Hummers’ procedure. The extensive peel-like feature in the SEM images of 8-min PAOM (Fig. 2d) further supports the significant oxidation, but it is not the case in 8-min Hummers’. In addition, the bulky analysis of XRD of the partially reacted samples is shown in Fig. 2e) with NaCl as internal standard. The patterns of 4-min samples under PAOM and Hummers are similar, while the appreciable difference can be observed between the 8-min comparisons. The larger d-spacing by 8-min PAOM suggests the higher quantity of Mn-oxidant consumption from the reaction mixture^7^, showing a good agreement with the EDS data. The evidence for faster diffusion in PAOM becomes evident, although the deep understanding of mechanism needs more systematic study.

**Figure 3:**
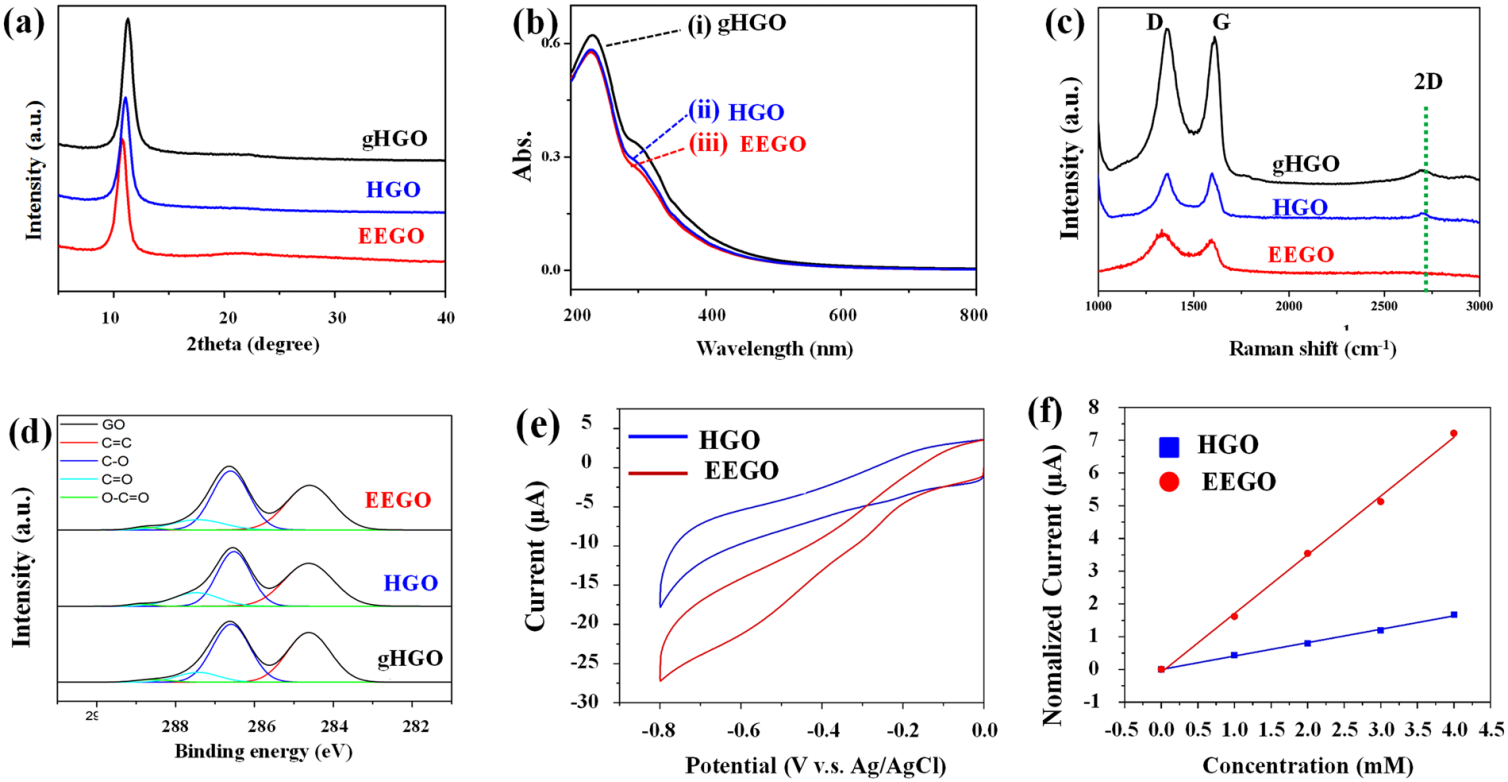
The characterization results and the electrochemical performance comparison of the GO products: (**a**) XRD patterns, (**b**) UV-vis, (**c**) Raman, and (**d**) XPS spectra. (**e**) The reduction current of EEGO and HGO acquired by CV in 5 mM H_2_O_2_. (**f**) The calibration curves of the amperometric responses corresponding to the CVs of EEGO and HGO in Fig. S-4.

The XRD patterns of all three GO products show the characteristic 2-theta peaks in the 10–12° region in Fig. 3(a), corresponding to the interlayer spacing of 0.841, 0.754, and 0.735 nm for EEGO, HGO, and gHGO, respectively. Because graphite oxidation induces the interlayer expansion^8^, the greatest interlayer spacing displayed by EEGO represents the highest oxidation level. In Fig. 3(b) the UV-vis spectra reveal an intense peak near 230 nm and a small shoulder in the 275–350 nm region, relevant to the *π*−π* transition of C=C and the *n*−π* transition of C=O functionality, respectively^19^. The gHGO gives the strongest absorption bands while EEGO and HGO show similar intensities at the same concentration, suggesting that the conjugation system is less disrupted in gHGO than the others, consistent with its insufficiently oxidized nature. In Fig. 3(c) even though the three GO samples unanimously exhibit the signature D (1350–1360 cm^−1^) and G bands (1592–1608 cm^−1^), the 2D band at 2695 cm^−1^ is only observable in gHGO and HGO (weaker). As the 2D band reflects the crystalline quality of graphitic and graphene materials^20^, the lack of this signal in EEGO implicates the destruction of the planar sp^2^ carbon network resulting from functionalization of the material. Furthermore, the greatest D-to-G ratios of EEGO (1.24) among the other two samples (1.05 for HGO and 1.03 for gHGO) indicate an abundance of structural defects, also ascribable to the high level of oxidation. The functional group identification by IR shows the vibration of -OH stretching (3000–3500 cm^−1^), C=O stretching (1720–1740 cm^−1^), C=C (1590–1620 cm^−1^), C-O (1250 cm^−1^), and C-O-C epoxy (1060 cm^−1^) in these GO samples, as labeled in Figure S-2 ^4^. The nearly identical IR spectra indicate the similar functional groups existing among these GO samples.

To quantitatively evaluate the relative oxidation degrees, we carried out the high-speed centrifuge (6000 rpm), then the carbon and oxygen contents, as well as the oxygen-to-carbon ratios of all three GO samples from the supernatant examined by surface-sensitive XPS and bulk EA. The precipitates (see Fig. S-3), due to incompletely oxidized graphite, were also collected and their weight percentages to the initial mass of graphite flakes are reported in Table 1. EEGO gave only a trace amount of precipitate (<0.2%), a signature of high oxidation degrees and monodispersity unparalleled by the others (2.0% for HGO and 8.6% for gHGO). In Fig. 3(d) deconvolution of the C1s XPS spectra illustrates various carbon species encompassing C=C (284.6 eV), epoxy/hydroxyls (C-O, 286.5 eV), carbonyl (C=O, 287.4 eV), and carboxylate (O-C=O, 288.9 eV), resonating well with the GO materials^12^. The fraction of the oxygenated carbon is 56.58% in EEGO, higher than those of HGO (54.30%) and gHGO (53.41%). Likewise, the EA results show the highest O/C ratio in EEGO (0.78), followed by HGO (0.72) and gHGO (0.65) (see Table 1). Nominally the O/C ratios do not seem significantly higher in EEGO, but the extent of oxidation when taken together with the amount of poorly oxidized graphite left behind in the precipitate would be much lower for HGO and gHGO.

**Table 1:** The EA and XPS data of the GO samples.

Samples	Mass ratios^a^ (precipitates)	Elemental analysis (EA)^b^ (supernatant)			XPS analysis^c^ (supernatant)				
		Oxygen contents	Carbon contents	O/C ratios	C-O (%)	C=O (%)	O-C=O (%)	C=C (%)	Oxidized carbon (%)^d^
EEGO	0.17%	3.00	3.85	0.778	40.76	14.71	1.11	43.42	56.58
HGO	2.0%	2.94	4.06	0.723	39.77	13.34	1.10	45.70	54.30
gHGO	8.6%	2.79	4.27	0.652	41.34	10.59	1.47	46.59	53.41
10X-EEGO	1.2%	2.96	4.08	0.725	39.18	14.87	2.30	43.65	56.35

^a^The ratios of un-exfoliated precipitate to the initial mass of graphite powders in the corresponding synthesis.

^b^The ratios of oxygen contents divided by carbon contents from EA.

^c^The percentages of each species are calculated based on the peak areas after deconvolution relative to that of the whole carbon signals.

^d^The ratios of the sum of all the oxidized carbon species versus the unoxidized carbon species (C=C).

Since the addition of KMnO_4_ needs to be slow in the conventional approach, it is problematic for scale-up preparation of GO. We tested the 10-time scale-up synthesis (10 g of graphite) by PAOM without extension of the reaction time, and the results were quite successful (see Fig. S-4). The GO resultants (denoted as 10X-EEGO) show an oxidation degree slightly lower than EEGO but still greater than HGO (see the comparisons of XPS and EA data in Table 1). In particular, with 10 times more graphite the centrifuge precipitate of 10X-EEGO occupies only 1.2%, lower than that for HGO. Although the time required for slow addition of 10X KMnO_4_ following the Hummers’ method is not exactly measured, PAOM represents an efficient and reliable option for highly oxidized GO preparation. The limit of scale up production has not been systematically tested, yet it should be capable of exceeding 10-time scale, considering the higher oxidation degrees of 10X-EEGO than HGO.

The level of oxidation was also found to influence the electrochemical biosensing of hydrogen peroxide. EEGO and HGO modified electrodes were fabricated and the cyclic voltammogram (CV) responses for a series of H_2_O_2_ with various concentrations in PBS solutions were recorded. As shown in Fig. S-5 (a) and (b), both electrodes demonstrate increased reduction peak current with the addition of H_2_O_2_. However, under the same H_2_O_2_ concentration (5 mM) the EEGO-modified electrode exhibits much better electrocatalytic activity towards H_2_O_2_ than HGO (Fig. 3(e)), substantiating that there is a correlation between the sensing performance and the oxidation degree of GO. The similar onset potential but distinct amperometric magnitude implies the electrocatalytic sites (or species) are the same but different in quantity^12^. We argue that the heavy oxidation causes the galleries to widen, thus making more active sites become accessible. In Fig. 3(f) the calibration curve from EEGO indicates a sensitivity of 1.794 μA/mM, 4.3 times greater than that (0.409 μA/mM) of HGO. Again GO with greater oxidation degree translates into the superior electrochemical activity.

**Figure 4:**
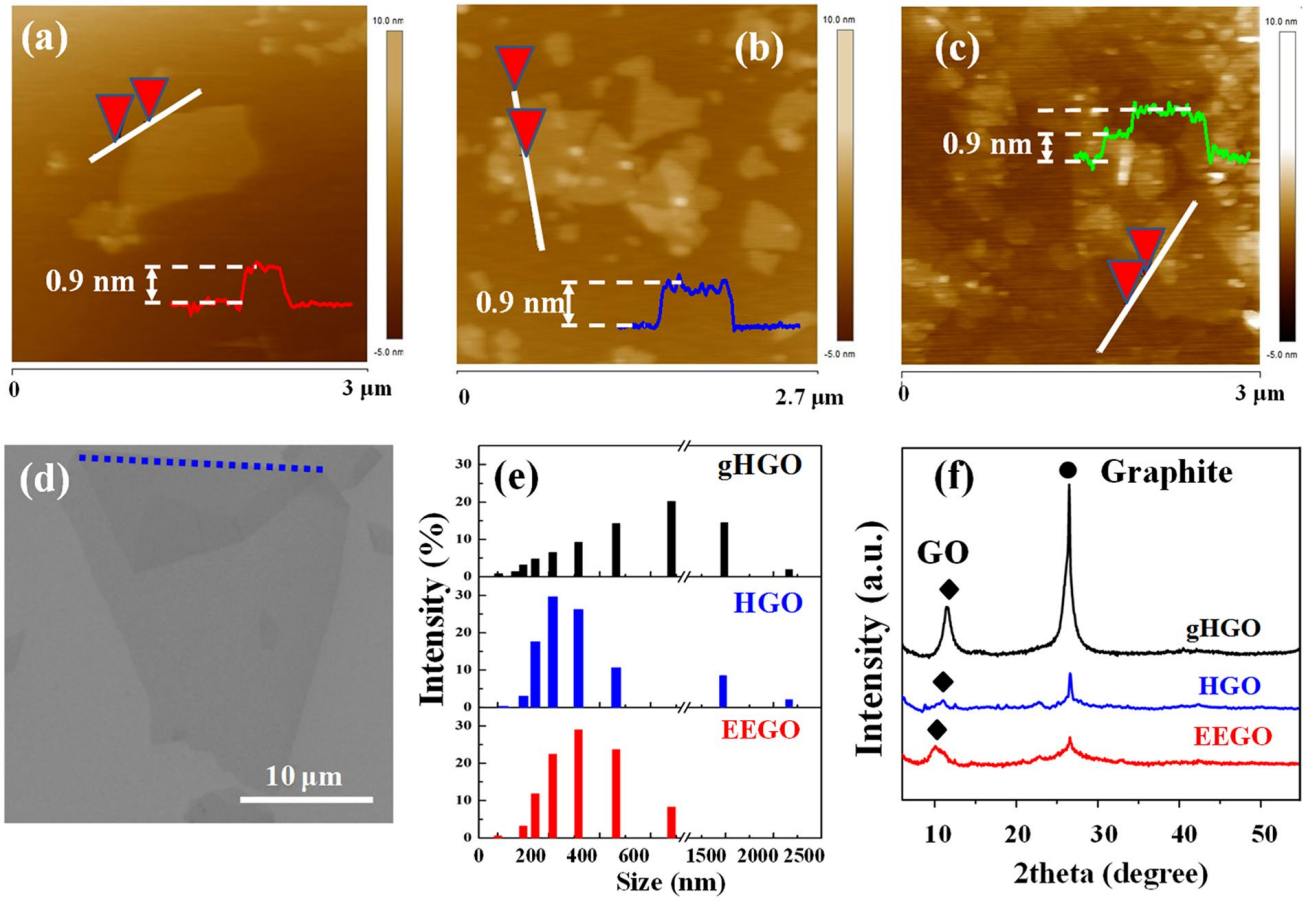
The AFM results of EEGO (**a**), HGO (**b**), and gHGO (**c**) products. The SEM images of an EEGO sheet (**d**) with a folding lines in blue. (**e**) The hydrodynamic size distribution of the GO samples by DLS. (**f**) The XRD of the precipitates sampled from each GO by a 6000 rpm centrifuge.

The physical dimensions of EEGO, HGO and gHGO nanosheets were analyzed by AFM. As shown in Fig. 4(a), the sheets produced by the PAOM method are more uniform with a thickness of 0.9 nm, consistent with the size for the single-layer GO^21^. In contrast, particle-like impurities are frequently observed on both HGO and gHGO in Fig. 4(b) and (c). Particularly, multiple-layer stacking can be identified on the gHGO sheets by the step-like features, indicative of the relatively poor exfoliation. Typical SEM images of EEGO platelets exhibit (see the example in Fig. 4(d)) large smooth 2D morphology with a size distribution of 22.5 ± 5.5 μm. Such the wide GO sizes (v.s. several microns of GO dimension commonly reported in the literature) can be valuable for transparent, conductive films^22^. The small GO sizes in AFM were acquired to fit the measurement dimension of AFM technique.

The complete exfoliation to the level of individual EEGO sheets, in conjunction with their highly decorated oxygen functionalities, yields stable and dispersed colloidal GO suspension in water. The DLS measurements were conducted, and the EEGO solution gives rise to the narrowest size distribution peaked at 300 nm whereas gHGO affords a much broader profile (up to 2100 nm) with the most probable size at 700 nm. HGO covers a size regime comparable to gHGO, yet its average peak size is similar to EEGO (see Fig. 4(e)). The XRD patterns of the centrifuge precipitates are shown in Fig. 4(f) where characteristic peaks of GO and graphite appear (labelled by diamonds and filled dots, respectively). The intensive graphite signal in gHGO is commensurate with its incomplete oxidation. Similar interlayer spacing resulting from the supernatant and the trace precipitate (Fig. 3(a) vs. 4(f)) of EEGO further warrants its feasibility to be deposited evenly onto substrates in thin film forms.

**Figure 5:**
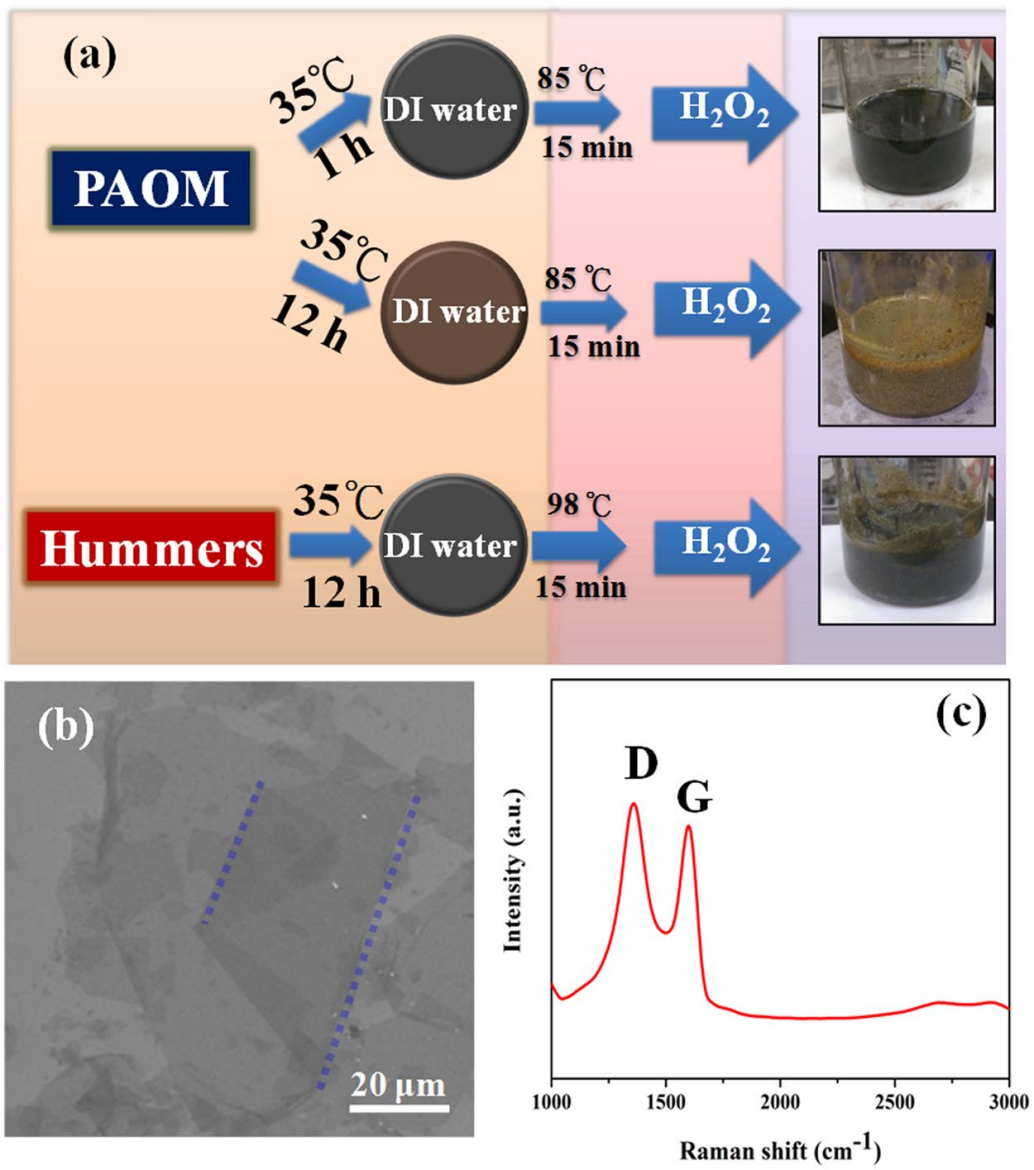
(**a**) The schematic procedure for oxidation of large size graphite flakes with varied reaction time of the 35 °C step. The SEM (**b**) and Raman (**c**) results of the GO products obtained by 12-h PAOM in (**a**). The blue dashed lines in (**b**) are folding lines of the individual GO sheet.

The strong oxidizing activity of PAOM can be potentially effective for exfoliation of bulky graphite crystals, particularly challenging for conventional Hummers’ method with strongly size-dependent reactivity^7^. Following the study above, we conducted both PAOM and the Hummers’ to oxidize the bulky 0.8 mm graphite crystals (Fig. 5). The products obtained by the PAOM at 35 °C for one hour show the insufficient exfoliation with dark green appearance with chunks similar to gHGO (the top pathway of Fig. 5a). As the 35 °C step has been recognized as the main oxidation stepα^7, 18^, we further extended the reaction time of 35 °C to 12 hours and resulted in the successful generation of yellow-brown GO (the middle pathway of Fig. 5(a)). The SEM images (Fig. 5(b)) show the average diameters of 65.4 ± 15.2 microns for individual GO sheets (with around 10% GO sheets wider than 100 μm). Such the GO dimensions are 3- to 4-time greater than that with 325-mesh graphite. The Raman data confirm the GO characteristics with D band stronger than G band. (Fig. 5(c)). In comparison, by extending the 35 °C step in Hummers’ method to 12 hours, only dark green products can be obtained (Fig. 5(a), the bottom pathway). We also tried to achieve the bulky graphite oxidization with tandem Hummers’ procedure, in which 35 °C step was conducted for 12 hours twice separately, but no success, emphasizing the mandatory one-shot oxidation for successful GO generation.

Under the same quantity of oxidizing reagents and graphite, both the syntheses should reach an equilibrium status after such the long time reaction (12 h). The drastic difference may be determined at the initial stage of the reactions. Except the concerns of Mn-oxidant diffusion into graphite galleries, the as-generated manganese oxide particles after redox with graphite may occupy the same diffusion paths for Mn-oxidants, and thus block further diffusion of the oxidants deeper into the graphite crystals. This phenomena will be more pronounced with increased diffusion lengths of larger sizes of graphite flakes exhibiting poor oxidation efficiency by the Hummers’ method. It is therefore that rapid diffusion of Mn-oxidants through individual graphite crystals at the initial stage, before the formation of blocking particles, is critical. On the basis of the experimental results above, PAOM exhibits the greater Mn penetration length and higher interlayer d-spacing at the beginning stage (within 8 min), playing the critical role in effective oxidation of bulky graphite. Although optimal preparation conditions have not been systematically investigated, PAOM represents the most promising solution to accomplish bulky GO nanosheets effectively. A comprehensive comparison of the reaction conditions and GO products of the Hummers-related approaches has been summarized in Table 2.

**Table 2:** The comparison of the Hummers-modified approaches and products.

Methods	Reaction time	Reaction temperature-I^b^	Reaction temperature-II^c^	O/C ratios	Average GO sizes
Hummers^5^	2–10 h^a^	35 °C	98 °C	0.444	—
Modified-1 (1999)^26^	8 h^a^	35 °C	98 °C	0.435	Several microns
Modified-2 (2004)^27^	5 days^a^	20 °C	—	0.556	20 μm
Modified-3 (2010)^8^	12 h^a^	50 °C	—	—	Several microns
Modified-4 (2011)^10^	24 h^a^	45 °C	45 °C	—	—
Modified-5 (2014)^28^	10–70 h^a^	45 °C	—	0.279–0.459	50 μm
Modified-6 (2015)^6^	1 h^a^	40 °C	95 °C	0.503	Several microns
Modified 7 (2015)^12^	16 h^a^	50 °C	—	—	Several microns
Modified-8 (2017)^29^	2 h^a^	35 °C	—	0.701	~10 μm
PAOM (This work)	1 h (The KMnO_4_ addition time included)	35 °C	85 °C	0.778	65.4 ± 15.2 μm

^a^The addition time of KMnO_4_ (slow addition) is not included.

^b^Reaction temperatures after H_2_SO_4_ mixing with graphite flakes.

^c^Reaction temperatures after adding DI water.

**Figure 6:**
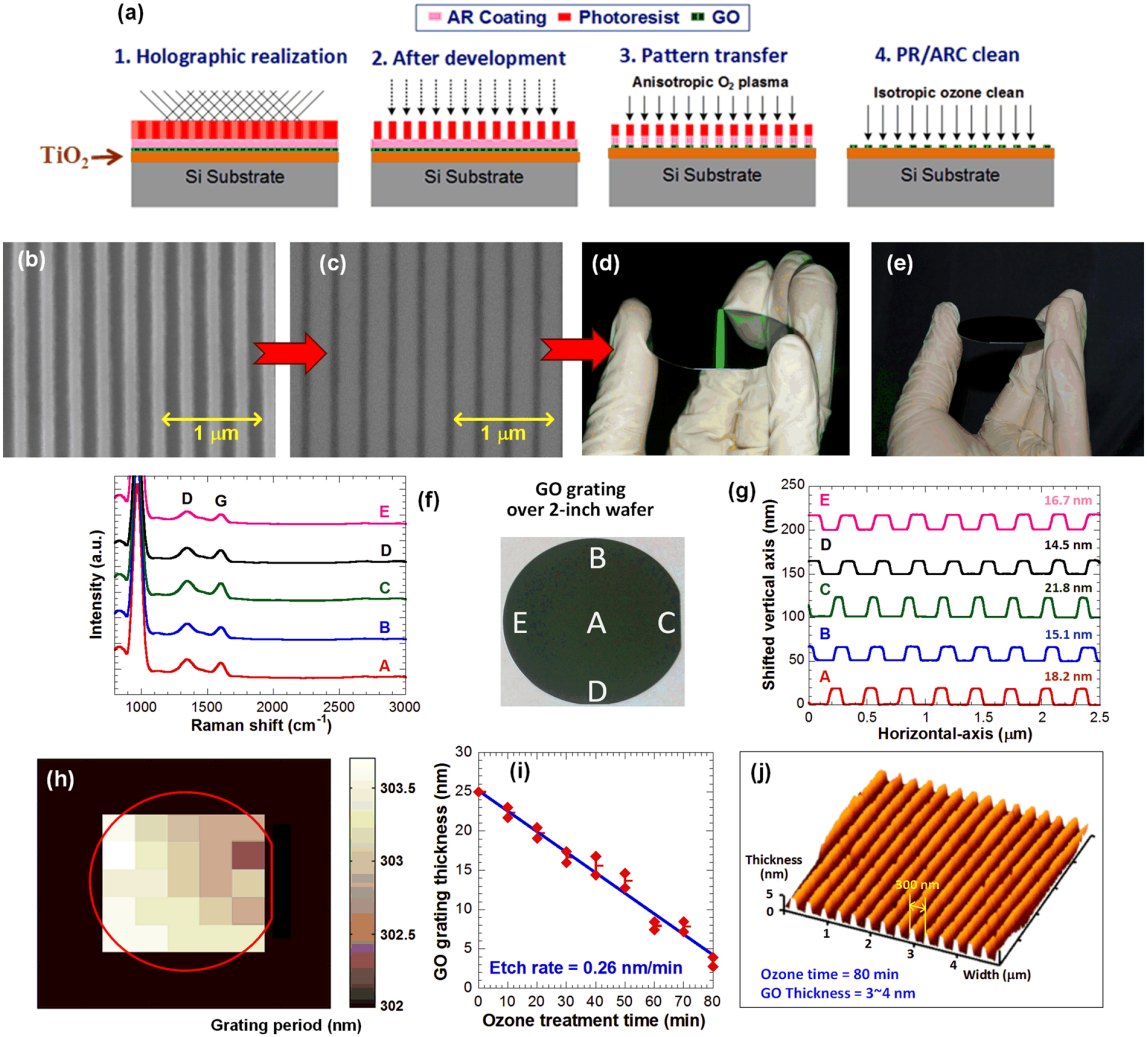
The wafer-scale nanopatterning of EEGO. The schematic illustration of patterning procedure (**a**).The SEM images of resist (**b**) and patterned EEGO after the removal of resist (**c**). The uniform light diffraction over the patterned EEGO on the wafer (**d**), compared to a bare 2-inch wafer (**e**). The Raman spectra (**f**) and AFM section analysis (**g**) correspond to the locations labelled by A to E in the middle inset on the wafer. (**h**) The full wafer mapping of the grating period. (**i**) The EEGO etching rates under ozone treatment and (**j**) AFM topography of the samples after 80 min etching.

Since films that can evenly cover the whole substrate surfaces with extremely low surface roughness (RMS ~0.590 nm, see Fig. S-6) are the requisite for nano-patterning at a wafer-scale, we made an attempt to fabricate large-area submicron GO-based periodic grating structures as the sternest challenge to colloidal EEGO and the procedure is depicted in Fig. 6(a). After spin coating the EEGO film, an antireflective coating (ARC), and a photoresist layer sequentially on a TiO_2_/Si substrate, the surface was exposed to an interference fringe produced by a laser holography lithography setup^23^, designed specifically for patterning over a large area with high throughput, to form a resist grating pattern which was then conveyed to the EEGO film by oxygen plasma etching. The residual resist/ARC was then cleaned by isotropic ozone treatment. According to the SEM images shown in Fig. 6(b) and (c), uniform resist pattern was loyally transferred into the EEGO film with a slightly undercut shrinkage, leading to the final gratings with a linewidth of 90 nm and a periodicity of 300 nm. The same process was successfully reproduced over a 2-inch wafer, as verified by the bright and uniform diffracted light (in green) observed in our end product (see Fig. 6(d)). No diffracted light can be observed on bare wafers under the identical illumination, confirming that the diffracted green light is due to the thin EEGO grating. The chemical composition and physical geometry of the realized EEGO gratings were tested for uniformity qualification based on Raman spectra and AFM profiles measured at five representative locations (A to E) chosen over the entire 2-inch wafer, together with mapping of the grating period by optical diffractometry. The presence of D- and G-band in all the Raman spectra, Fig. 6(f), indicates that the GO characteristics were universally preserved. In Fig. 6(g) the AFM section analysis reveals consistent grating depths with variations less than ~7 nm, of which the cause is known to be instrumental, a consequence of the Gaussian distribution inherent to the laser beam of the interference system^24^. The mapping in Fig. 6(h) exhibits a well-defined periodicity across the whole wafer range with only ~1 nm variations. Here we demonstrate that the isotropic ozone treatment can be utilized to etch the periodic EEGO structure to the desired thickness without compromising on the quality of the product (Figure S-7). When complemented with the exceptional smoothness of the EEGO film, the slow 0.26 nm/min etch rate (evidenced in Fig. 6(i)), equivalent to ~1/3 of GO monolayer per minute, provides flexible and precise thickness determination of the final gratings. The AFM topography shown in Fig. 6(j) clearly displays that after 80 min ozonation^25^, the height of the GO gratings was adjusted to as small as 4 nm that can permit very little tolerance for film non-uniformity.

## Conclusion

In this work we have successfully demonstrated that the PAOM approach is an effective and efficient GO preparation method capable of producing highly oxidized GO in a large quantity. The elevated contents of oxidized carbon, which serve as the chemical derivatization sites and carry repulsive charges for improved dispersion, enable a wide utilization of EEGO as starting materials for graphene derivatives in further chemistry-related tasks. In terms of engineering aspect, EEGO features complete exfoliation, great yields, and uniform dispersion into water, making high throughput production of wafer-scale graphene nanopatterns feasible and industrial-ready. The precise controls of lateral width and thickness of GO in nanopatterning provide the new roadmaps of graphene in 2D array quantum dots, chemical sensors, wearable electronics, bendable optical gratings, and many others.

## Electronic supplementary material

Supplementary Information
