# Supplementary material for: Effective Synthesis of Highly Oxidized Graphene Oxide That Enables Wafer-scale Nanopatterning: Preformed Acidic Oxidizing Medium Approach
Source: Sci Rep. 2017 Jun 20;7:3908. doi: 10.1038/s41598-017-04139-0 (PMC5478619; doi:10.1038/s41598-017-04139-0)

Supplementary information for

Effective Synthesis of Highly Oxidized Graphene Oxide That Enables Wafer-scale Nanopatterning: Preformed Acidic Oxidizing Medium Approach

Chun-Hu Chen,[a*] Shin Hu,[a] Jyun-Fu Shih,[b] Chang-Ying Yang,[a] Yun-Wen Luo,[a] Ren-Huai Jhang,[a] Chao-Ming Chiang,[a*] and Yung-Jr Hung[b*]

[a]Department of Chemistry, [b]Department of Photonics, National Sun Yat-sen University, Kaohsiung, Taiwan 80424

Figure S-1. The photographs of 325-mesh graphite (left) and 0.8 mm graphite (right), respectively. The later one is the starting material for the diffusion study.

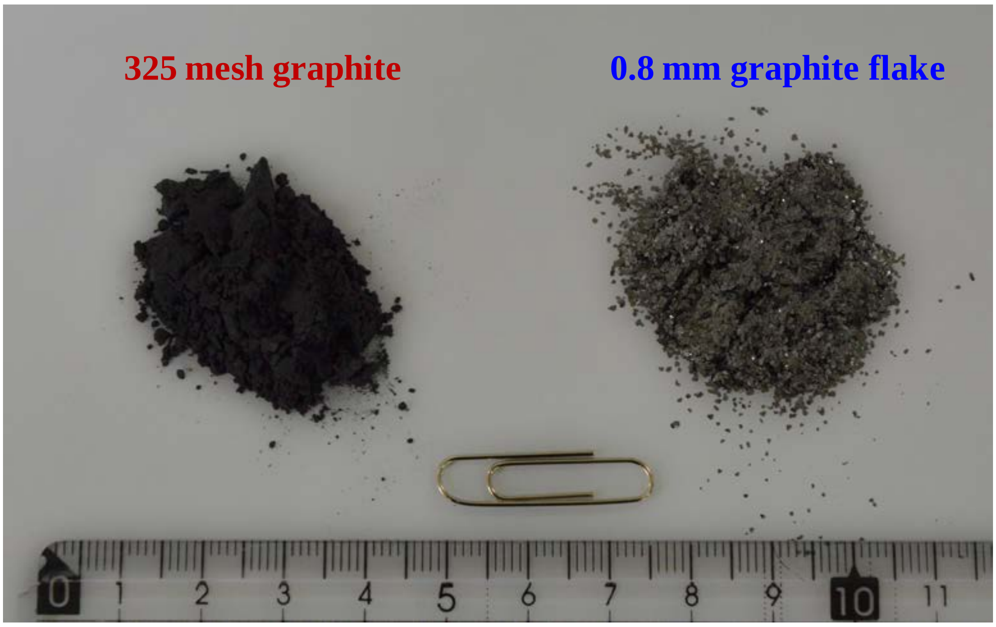

Figure S-2. The IR comparison of the GO samples. The -OH stretching (3000-3500 cm^{-1}, the pink area), C=O stretching (1720-1740 cm^{-1}, the blue area), C=C (1590-1620 cm^{-1}, the purple area), C-O (1250 cm^{-1}, the orange area), and C-O-C epoxy (1060 cm^{-1}, the green area) are observed in these GO products.

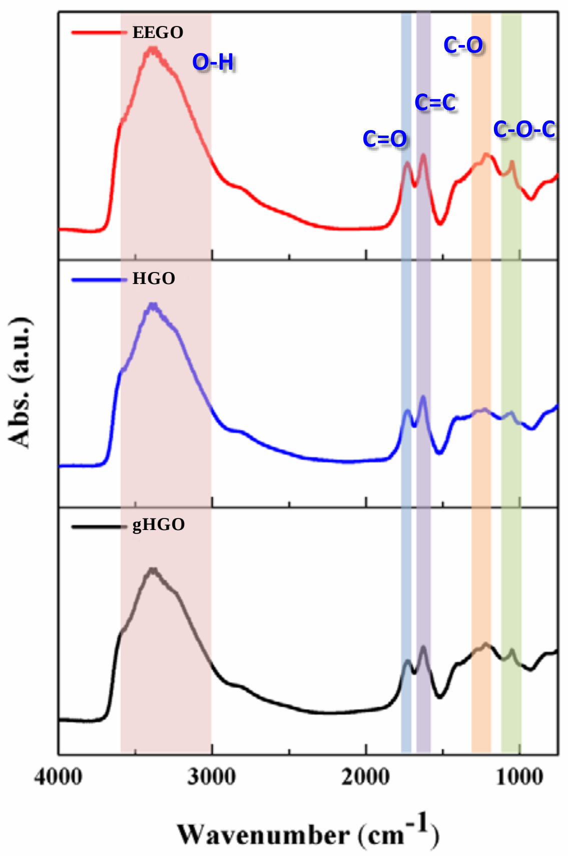

Figure S-3. The comparison of black precipitation collected after the high-speed centrifuge of 6000 rpm. The trace amount of precipitation in EEGO clearly shows the greatest dispersion homogeneity among all the other samples, corresponding well to the DLS results.

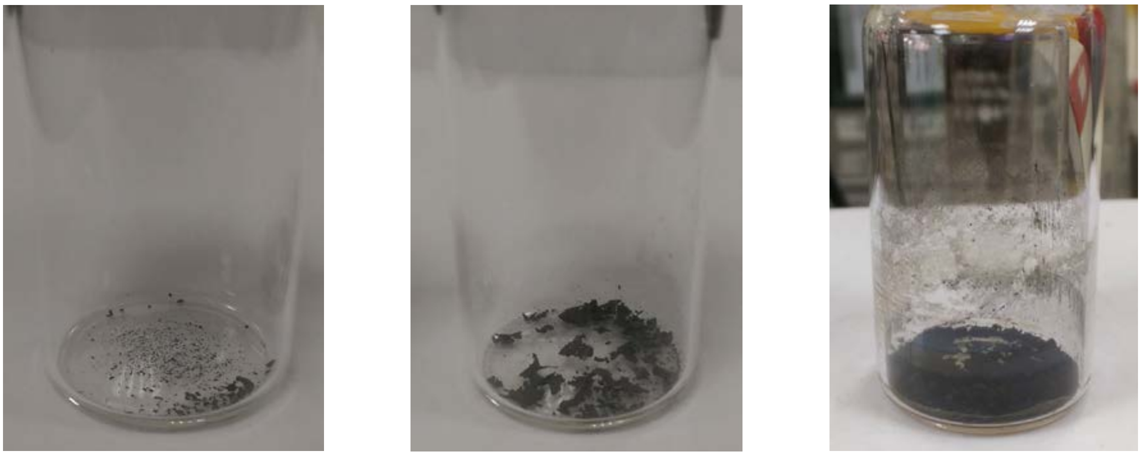

EEGO HGO gHGO

Figure S-4. The photographs (a-b) and XPS results (c) of 10X-EEGO prepared by PAOM within 1-2 hours.

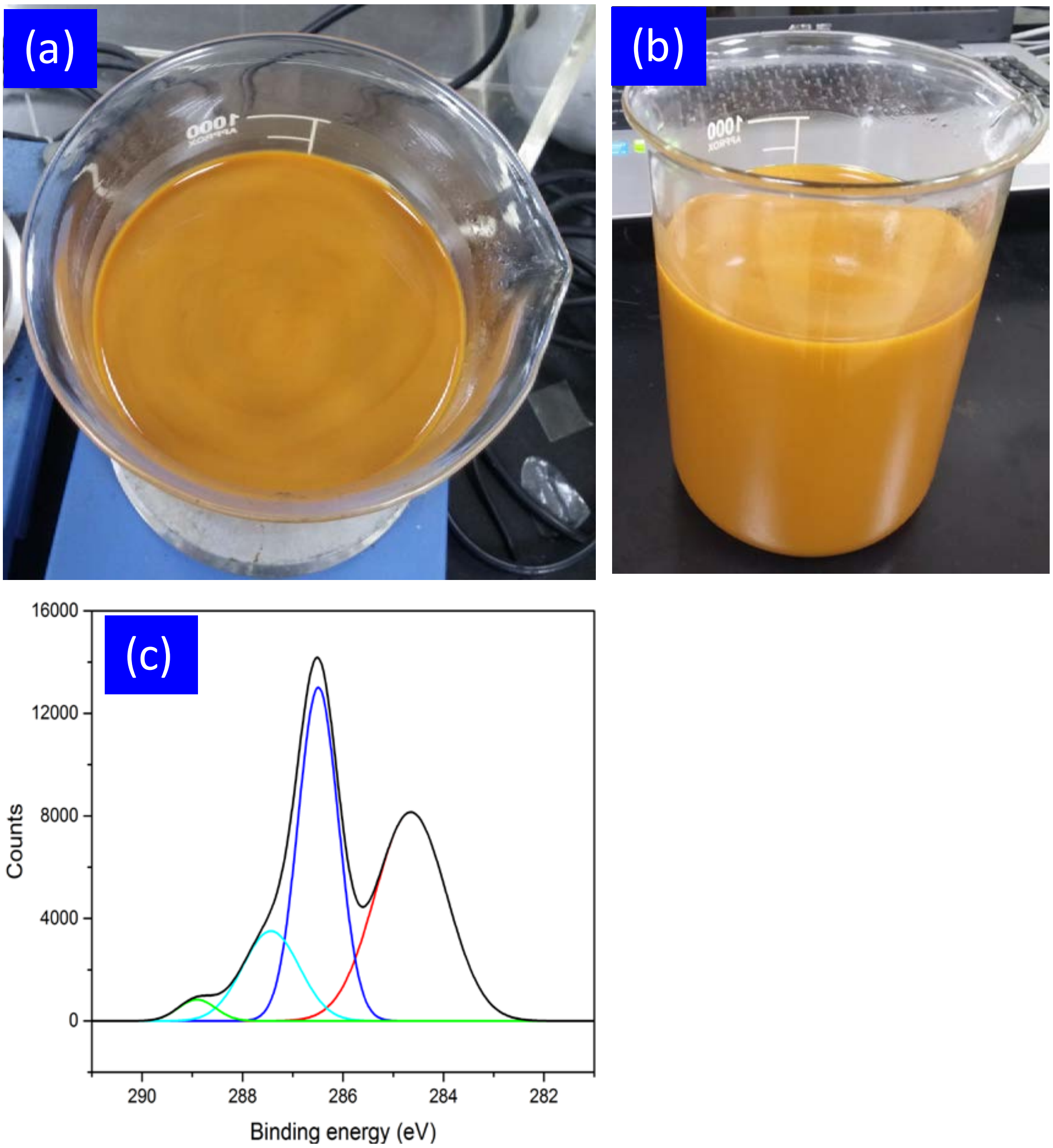

Figure S-5. The comparison of electrochemical sensing performance toward H_2O_2 in PBS. The cyclic voltammogram (CV) results of EEGO (a) and HGO (b) in the presence of H_2O_2 over a concentration range from 1.0 to 5.0 mM at a scan rate of 50 mV s^{-1}.

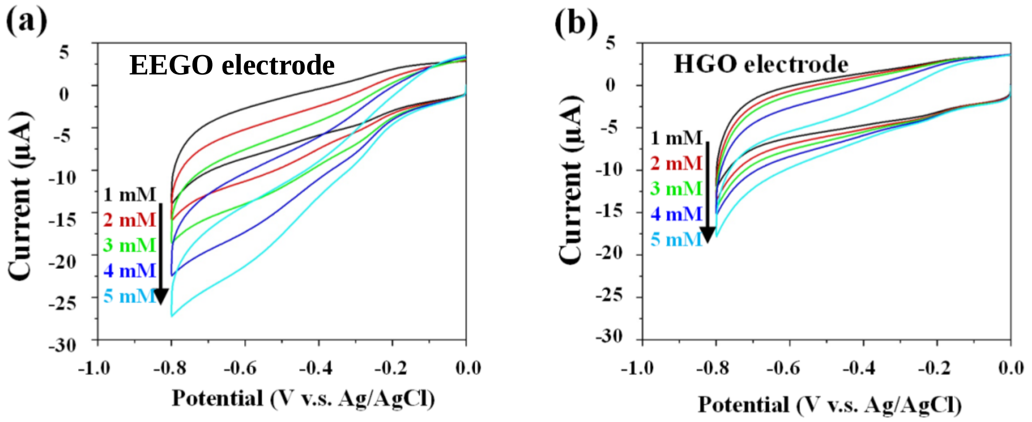

Figure S-6. The AFM results of spin-coat EEGO films on 2-inch wafer before patterning. The average RMS value of various wafer areas (A-E of Fig. 6) is 0.590 nm.

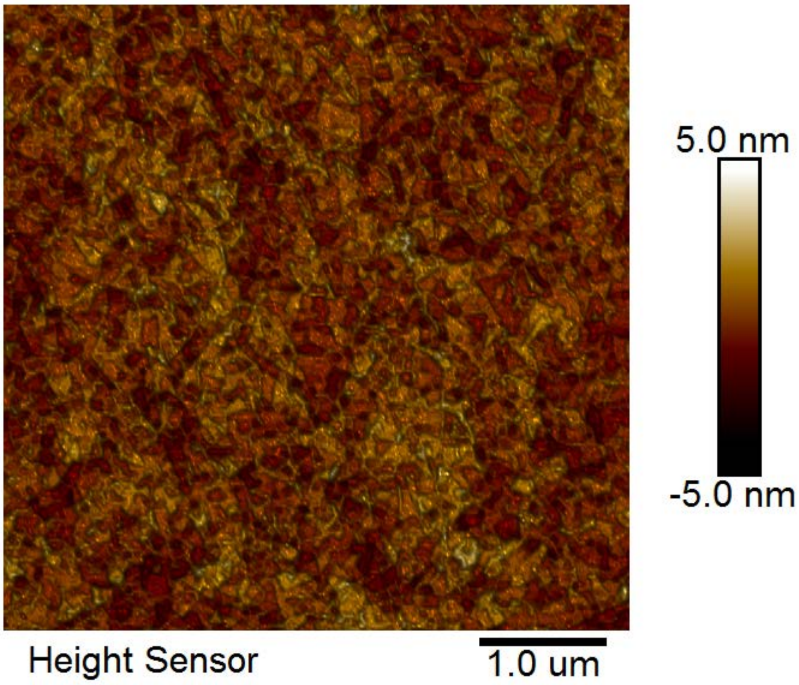

Figure S-7. Raman spectra of (a) spherical GO patterns with two-dimensional GO array (period = 10 µm). The Raman spectra demonstrate that the GO material can be completely removed after oxygen plasma etching due to the absence of characteristic D and G bands of GO outside the GO pattern. This results verify the generation of discontinuous GO nanopatterning.

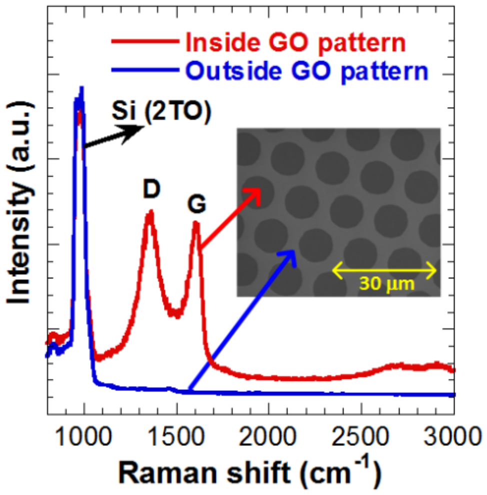

Supplement: Supplementary file 1 — Supplementary Information [file 41598_2017_4139_MOESM1_ESM.pdf]
